# Prevalence and Predictors of Falls Among Younger and Older Adult Pilgrims During the Hajj Mass Gathering: An Age-Stratified Cross-Sectional Study

**DOI:** 10.3390/jcm14217775

**Published:** 2025-11-02

**Authors:** Hammad Alhasan, Mansour Abdullah Alshehri

**Affiliations:** Department of Medical Rehabilitation Sciences, Faculty of Applied Medical Sciences, Umm Al-Qura University, Mecca 24382, Saudi Arabia; mamshehri@uqu.edu.sa

**Keywords:** fall risk, younger adults, older adults, obesity, hypertension, diabetes, physical exhaustion, musculoskeletal pain

## Abstract

**Background/Objectives**: Hajj is a physically demanding mass gathering that presents distinct health risks, particularly for older adults and individuals with comorbidities. Falls are a major cause of injury in such environments; however, limited data exist on their prevalence and determinants during Hajj. This study aimed to (1) estimate the prevalence of falls among adult pilgrims during the Hajj pilgrimage in Saudi Arabia and (2) identify key demographic, behavioural/clinical, and musculoskeletal predictors of fall risk, stratified by age group. **Methods**: A cross-sectional survey was conducted among 1429 adult pilgrims. Data were collected at major pilgrimage sites in Mecca during the Hajj season. Variables included age, sex, body mass index, smoking status, hypertension, diabetes, physical exhaustion, and musculoskeletal pain. Bivariate chi-square tests and multivariable regression analyses were performed. Age-stratified models were developed for younger adults (≤29 years) and older adults (≥50 years) to account for physiological differences. **Results**: The overall fall prevalence was 13.6%, with significantly higher rates among older adults (21%) than younger adults (10.5%). In the full sample, independent predictors of falls included older age, obesity, hypertension, diabetes, physical exhaustion, and musculoskeletal pain in the upper arm, elbow, and hip/pelvis. In age-specific models, obesity, physical exhaustion, and upper arm pain predicted falls among younger adults, while obesity, hypertension, physical exhaustion, and hip/pelvis pain were significant among older adults. **Conclusions**: Falls during Hajj result from a multifactorial interplay of age, comorbidities, fatigue, and site-specific musculoskeletal pain. These findings support the development of targeted, age-specific fall prevention strategies in mass gathering contexts.

## 1. Introduction

Hajj is the Arabic term for pilgrimage, a religious obligation that every Muslim must perform at least once in their lifetime if they possess the necessary physical and financial means, as it constitutes one of the five pillars of Islam [1,2,3]. Hajj involves a set of rituals performed over five days in Mecca and its surrounding holy sites, during which more than 1.8 million pilgrims gather annually in one of the world’s largest mass gatherings [4]. These rituals require significant physical exertion, including walking long distances, ranging from 5 to 15 km (approximately 3 to 9 miles) daily, with an estimated cumulative distance of 63 km (about 39 miles) over the pilgrimage period [5,6]. Pilgrims are also required to repeatedly stand, walk, bend, and carry personal belongings, further increasing physical demand [7]. These conditions, coupled with extreme environmental heat, pose significant health challenges for both pilgrims and health authorities [1,8].

Pilgrims participating in Hajj represent a wide range of nationalities, ages, and health conditions [4,9]. A substantial proportion are over the age of 50 and often suffer from clinical health diseases such as diabetes, hypertension, and musculoskeletal pain, which can compromise their safety and endurance during the pilgrimage [10,11]. Physical exertion becomes especially hazardous for older adults with these comorbidities, increasing their risk of injury, including musculoskeletal trauma and falls [12,13]. The risk is further exacerbated by the high summer temperatures during recent Hajj seasons, with temperatures reaching up to 50 °C. These conditions heighten the risk of dehydration, heatstroke, and subsequent physical instability, particularly in individuals with diabetes and hypertension [8].

Falls represent a major global public health concern among older adults, with approximately 27% affected annually [14]. According to the World Health Organization (WHO), falls are the second leading cause of unintentional injury-related deaths worldwide, especially among individuals aged 60 and above [15]. Beyond mortality, falls can result in serious injuries such as fractures and head trauma, and are associated with immobility, hospitalization, and long-term disability [16,17]. Psychological effects, including fear of falling and loss of confidence, further contribute to reduced activity and quality of life [17,18]. These consequences are particularly concerning in the context of Hajj, where rapid access to emergency medical care may be delayed due to crowd density and logistical barriers. The WHO has also reported a considerable burden of injuries during Hajj, including those caused by falls [15].

Despite the extensive literature on fall-related risk factors in older adults, there is a notable lack of studies focusing on falls specifically during Hajj. The pilgrimage setting presents a unique combination of physical, environmental, and psychosocial stressors that can amplify fall risk, particularly in older adults with clinical health conditions and functional limitations. Previous research has identified several fall-related risk factors, including increasing age, high body mass index (BMI), smoking or comorbidities such as diabetes and hypertension, and the presence of musculoskeletal pain [19,20,21,22,23,24,25,26]. Multisite pain, including in the back and lower extremities, has been shown to increase fall risk significantly [27]. Frailty, a common geriatric syndrome associated with chronic illness, is another key contributor to fall susceptibility [28,29]. Other contributing factors include fatigue, muscle weakness, impaired balance, and decreased functional mobility [30,31,32].

Although several studies have reported injury patterns during the Hajj pilgrimage, falls have rarely been examined as a distinct clinical outcome. Most existing research has focused on general trauma, fractures, or musculoskeletal conditions, without specifically addressing the prevalence of falls or identifying their predictors [33,34]. For example, Alghamdi et al. [13] described musculoskeletal injuries among pilgrims but did not identify or classify fall-related incidents. Similarly, Alshehri et al. [6] reported widespread musculoskeletal pain during Hajj, yet did not investigate its potential association with falls. Comprehensive literature reviews by Aldossari et al. [10] and Madani et al. [3] acknowledged the role of falls in contributing to hospital admissions and injury burden; however, their analyses lacked detail regarding specific risk factors or demographic trends [3,4]. Furthermore, the WHO [15] has recognized falls as a leading cause of injury globally and in mass gatherings, but no study has systematically explored age-specific risk factors for falls during Hajj [5]. This gap is particularly concerning given the increasing number of older pilgrims and the physical demands of the pilgrimage. Identifying age-related risk factors is therefore essential for informing targeted public health strategies and preventive education. This study addresses this gap by providing an epidemiological analysis of fall prevalence and its demographic, clinical, and musculoskeletal determinants among pilgrims, with a specific focus on age-related differences in risk.

To date, no studies have comprehensively examined the prevalence or predictors of falls among Hajj pilgrims, nor have they explored how demographic and medical factors interact within this specific mass gathering environment. This gap limits the development of preventive strategies and health guidelines tailored to the needs of pilgrims. Given the aging global Muslim population and the growing number of older adults undertaking Hajj, addressing fall risks in this context is both timely and essential, locally for public health planning in Saudi Arabia, and globally for informing the health preparedness of all participating countries. This study aimed to: (1) estimate the prevalence of falls among adult pilgrims during Hajj; and (2) identify key predictors of falls, including sex, BMI, smoking status, physical exhaustion, comorbidities (e.g., diabetes and hypertension), and musculoskeletal pain among younger and older adult pilgrims. The primary goal of this study is to produce epidemiological evidence that can guide public health strategies for fall prevention during mass gatherings, by identifying high-risk profiles through age-stratified analyses. The results may also have secondary utility for clinical risk stratification, particularly in pre-travel assessments and triage planning.

## 2. Materials and Methods

### 2.1. Study Design

A cross-sectional survey was conducted among pilgrims during the Hajj season using convenience sampling. Ethical approval was obtained from the Ethics Committee, Faculty of Applied Medical Sciences, Umm Al-Qura University, Mecca, Saudi Arabia. The study adhered to the ethical principles of the Declaration of Helsinki and followed the Strengthening the Reporting of Observational Studies in Epidemiology (STROBE) guidelines.

### 2.2. Participants

Participants were adult pilgrims (aged ≥ 18 years) of all nationalities who had completed the Hajj pilgrimage. Participants who had performed Umrah only were excluded, as Umrah involves a shorter and less physically demanding set of rituals. Furthermore, persons engaged in providing services to pilgrims (such as transport workers, safety officers, social and medical support staff) were excluded if they had not themselves completed the full Hajj rites. Informed consent was obtained from all participants. Participation was voluntary, and anonymity and confidentiality were ensured throughout the data collection and analysis process.

### 2.3. Data Collection

Data collection was conducted following the second day of Hajj, during the period from 21 to 31 August 2018. Data were collected during the Hajj season, between the initial and final days of the rituals, at key pilgrimage sites across Mecca, including Mina, Muzdalifah, and the Holy Mosque. Trained healthcare professionals, who had received instruction on standardized data collection procedures, approached pilgrims using either paper-based or digital (tablet/mobile) versions of the survey. Participants could complete the survey on their own or with the assistance of trained data collectors who offered clarification when needed. The digital version of the survey was hosted on a validated platform (www.surveymonkey.com). Paper forms were later transcribed into the same system by the research team to create a unified dataset. To enhance data accuracy and reduce transcription errors, each paper-based response was entered into the electronic survey system by a trained research assistant and independently cross-checked by a second reviewer. Any discrepancies were resolved by consulting the original paper forms. This double-entry verification process ensured consistency and reliability across data formats. All data were securely stored in a password-protected master file accessible only to the authors. While formal response rate was not systematically recorded due to logistical constraints at the pilgrimage sites, all data collectors followed a standardized protocol to ensure consistent administration. Participants requiring assistance received support from trained healthcare professionals who adhered to uniform phrasing and instructions. However, inter-rater reliability was not formally tested.

### 2.4. Survey

The structured survey, available in both Arabic and English, was designed to minimize response bias by collecting data in a sequential manner. It encompassed three main domains: (1) Demographic and behavioural/clinical variables, including age, sex, BMI (calculated from self-reported height and weight), smoking status, hypertension, diabetes, and physical exhaustion; physical exhaustion was assessed using a single yes/no question: “Did you feel physically exhausted during the Hajj rituals?”. (2) Fall events, where participants were asked whether they had experienced a loss of balance resulting in a fall during Hajj. (3) Musculoskeletal pain, with participants indicating any pain or discomfort experienced during Hajj and specifying the anatomical sites affected, allowing for multiple selections. All survey data were self-reported by participants through perception-based questions rather than standardized or validated scales, reflecting their individual experiences and interpretations during the Hajj rituals.

### 2.5. Sample Size Calculation

According to a 2024 report by the Saudi General Authority for Statistics, approximately two million individuals were expected to perform the Hajj pilgrimage. Based on this estimated population size, the required sample size was calculated using a 99% confidence level and a 5% margin of error, yielding a minimum of 666 participants. A pragmatic convenience sampling approach was adopted, appropriate for operational research in the Hajj setting where probabilistic sampling was not feasible. Participants were recruited across multiple sites and time windows using a standardized script to minimize recruitment bias. The a priori target of 666 participants represented the minimum required for adequate precision of the primary outcome, and recruitment was extended to 1429 participants to enhance estimate precision and support age-stratified analyses.

### 2.6. Statistical Analysis

For the stratified analysis, two distinct age groups were examined: younger adults (≤29 years) and older adults (≥50 years). This approach was selected to reflect the potential physiological and functional contrasts between the youngest and oldest participants. Younger adults are generally less affected by chronic conditions and may exhibit greater physical capacity, whereas older adults are more likely to experience fatigue, balance challenges, and comorbidities that could increase fall susceptibility. The intermediate 30–49-year group was not included in the stratified models to reduce within-group variability and to maintain clearer analytic contrast between the two age extremes. This approach may help to identify potential age-related differences in fall risk during the Hajj and guide future studies toward more refined age-specific analyses.

Data analysis was performed using IBM SPSS Statistics for Windows, Version 27. A two-tailed *p*-value of less than 0.05 was considered statistically significant for all analyses.

First, descriptive statistics, including frequencies and percentages, were used to summarize participants’ characteristics and other related information. These included demographic variables (sex and BMI), behavioural and clinical health conditions (smoking status, hypertension, and diabetes), physical exhaustion, fall prevalence, and the presence of musculoskeletal pain within each age group (≤29 years and ≥50 years). Prevalence rates were calculated and reported with corresponding 95% confidence intervals (CIs). Chi-square tests were used to compare the prevalence of these variables between the two age groups.

Second, descriptive statistics were used to summarize the same variables across fall status groups (fallers and non-fallers). Chi-square tests were applied to examine bivariate associations between fall prevalence and participants’ demographic characteristics, behavioural and clinical health conditions, physical exhaustion, and musculoskeletal pain. Prevalence rates were also reported with 95% CIs.

Third, multivariable binary logistic regression analyses were performed to identify significant predictors of falls. Separate models were developed for the overall sample as well as for each age group (≤29 years and ≥50 years). Independent variables included sex, BMI, smoking status, hypertension, diabetes, physical exhaustion, and the presence of musculoskeletal pain. The decision to stratify the regression analyses by age group was consistent with the study’s primary objective of investigating fall prevalence and associated risk factors in two distinct adult populations with differing physiological and functional profiles. Younger adults are typically characterized by greater physical resilience and lower fall risk, whereas older adults are more prone to falls due to age-related declines in muscle strength, balance, and general health. The age-specific models were designed to determine whether the pattern of predictors varied between younger and older adult pilgrims. Results were reported as regression coefficients (B) with standard errors (SE), along with odds ratios (ORs) and corresponding 95% CIs.

## 3. Results

**Figure 1:**
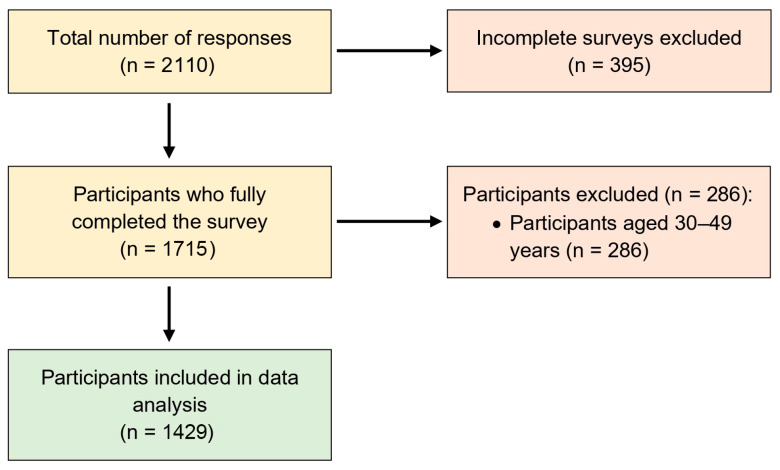
Flowchart of study participants.

A total of 2110 responses were received. Of these, 1715 (81.3%) contained complete information across all survey sections. Among the complete responses, 1429 (83.3%) met the predefined eligibility criteria for the study age groups. Figure 1 presents the flowchart of study participants. Overall, 195 participants (13.6%) reported experiencing a fall during the Hajj. The prevalence of falls was higher among older adults (27.1%, 87 of 321) compared with younger adults (11.8%, 108 of 913), highlighting potential age-related differences in fall risk that were further examined in subsequent analyses.

### 3.1. Characteristics of Younger and Older Adult Pilgrims

A total of 1429 participants were included in the analysis, comprising 1021 (71.5%) younger adults (aged ≤ 29 years) and 408 (28.5%) older adults (aged ≥ 50 years). Significant differences in demographic, clinical, and musculoskeletal variables were observed between the two age groups. Older adults had a significantly higher prevalence of obesity (BMI ≥ 30 kg/m^2^) compared to younger adults (43% vs. 21.1%, *p* < 0.001). Similarly, the prevalence of hypertension (43% vs. 6.5%, *p* < 0.001) and diabetes (33% vs. 6%, *p* < 0.001) was markedly higher among older adults. Physical exhaustion was also more commonly reported by older adults than younger adults (56.4% vs. 40%, *p* < 0.001). In terms of musculoskeletal pain, older adults reported a significantly higher prevalence of overall musculoskeletal pain (88% vs. 79%, *p* < 0.001). Specific pain sites that were significantly more common among older adults included the elbow (5% vs. 2%, *p* = 0.010), forearm (5% vs. 2.5%, *p* = 0.006), wrist/hand (8% vs. 3.5%, *p* < 0.001), hip/pelvis (11% vs. 4%, *p* < 0.001), knee (40.5% vs. 15.5%, *p* < 0.001), and leg (35.5% vs. 28.5%, *p* = 0.009). Table 1 presents the full descriptive analysis comparing younger and older adult pilgrims.

### 3.2. Bivariate Chi-Square Analysis of Fall Prevalence

Chi-square analyses revealed several statistically significant associations between participant characteristics and fall prevalence. Sex was significantly associated with falls, with a higher prevalence among females compared to males (16.1% vs. 11.6%, *p* = 0.015). Age group was also significantly related to fall prevalence, with older adults (≥50 years) reporting a higher fall prevalence than younger adults (≤29 years) (21% vs. 10.5%, *p* < 0.001). Although smoking status was not significantly associated with falls, smokers showed a higher prevalence than non-smokers (15.5% vs. 13.1%, *p* = 0.318). Among clinical conditions, participants with hypertension had a higher fall prevalence compared to those without (27% vs. 11%, *p* < 0.001), and a similar pattern was observed for diabetes (28% vs. 72%, *p* < 0.001). Physical exhaustion was also significantly associated with falls (21% vs. 11%, *p* < 0.001). In terms of musculoskeletal pain, participants reporting any musculoskeletal pain had a significantly higher fall prevalence compared to those without (16% vs. 84%, *p* < 0.001). Several specific musculoskeletal pain sites were significantly associated with fall prevalence, including the shoulder (21%, *p* < 0.001), upper arm (35%, *p* < 0.001), elbow (38%, *p* < 0.001), forearm (26%, *p* = 0.011), wrist/hand (29%, *p* < 0.001), head (19%, *p* = 0.009), cervical spine (20.5%, *p* = 0.001), thoracic spine (20%, *p* = 0.002), hip/pelvis (30%, *p* < 0.001), and knee (18%, *p* = 0.003). Table 2 presents the results of chi-square analyses examining the bivariate associations between all adult pilgrims’ characteristics and fall prevalence.

### 3.3. Multivariable Logistic Regression Analysis of Predictors of Fall Prevalence

Both logistic regression models demonstrated good overall fit, as indicated by non-significant Hosmer–Lemeshow tests (*p* > 0.05), suggesting that the predicted probabilities aligned well with observed outcomes in both age groups. Model 1 (Younger Adults): Explained 30.6% of the variance in falls, indicating moderate predictive power. Model 2 (Older Adults): Explained 33.6% of the variance in falls, also reflecting moderate strength. In both models, no evidence of multicollinearity was detected (all VIFs < 2 and tolerances > 0.1), indicating that the included predictors did not distort the regression coefficients.

Multivariable logistic regression analysis revealed several significant predictors of fall prevalence among all adult pilgrims. Older age (≥50 years) was associated with significantly higher odds of falling compared to younger age (≤29 years) (OR = 2.11, 95% CI: 1.57–2.82, *p* < 0.001). Obesity (BMI ≥ 30 kg/m^2^) was also positively associated with fall risk (OR = 1.84, 95% CI: 1.26–2.69, *p* = 0.002). Participants with hypertension (OR = 2.31, 95% CI: 1.54–3.45, *p* < 0.001) and diabetes (OR = 1.90, 95% CI: 1.24–2.91, *p* = 0.003) had significantly greater odds of experiencing a fall. Physical exhaustion emerged as a particularly strong predictor (OR = 2.83, 95% CI: 1.96–4.07, *p* < 0.001). In terms of musculoskeletal pain, upper arm pain (OR = 2.16, 95% CI: 1.03–4.56, *p* = 0.042), elbow pain (OR = 4.13, 95% CI: 2.25–7.57, *p* < 0.001), and hip/pelvis pain (OR = 2.10, 95% CI: 1.17–3.79, *p* = 0.013) were significantly associated with increased fall risk, whereas lumbar pain was inversely associated (OR = 0.63, 95% CI: 0.42–0.94, *p* = 0.023). Table 3 presents the results of the multivariable logistic regression analysis identifying predictors of fall prevalence among all adult pilgrims.

Among younger adult pilgrims, multivariable logistic regression analysis identified several significant predictors of falls. Obesity (BMI ≥ 30 kg/m^2^) was associated with greater odds of falling (OR = 2.05, 95% CI: 1.06–3.95, *p* = 0.033). Physical exhaustion showed a strong association with fall prevalence (OR = 4.66, 95% CI: 2.10–10.36, *p* < 0.001). Additionally, upper arm pain was a significant musculoskeletal predictor, with affected individuals having markedly higher odds of falling (OR = 12.26, 95% CI: 2.06–72.90, *p* = 0.007). Table 4 summarizes the results for younger adult pilgrims (aged ≤ 29 years).

Among older adult pilgrims, multivariable logistic regression analysis identified multiple significant predictors of falls. Obesity (BMI ≥ 30 kg/m^2^) was positively associated with fall risk (OR = 2.24, 95% CI: 1.31–3.85, *p* = 0.003). Hypertension also emerged as a significant predictor (OR = 2.28, 95% CI: 1.26–4.13, *p* = 0.010), as did physical exhaustion (OR = 2.40, 95% CI: 1.56–3.70, *p* < 0.001). Among musculoskeletal factors, hip/pelvis pain was significantly associated with increased fall risk (OR = 2.66, 95% CI: 1.23–5.74, *p* = 0.013), whereas lumbar pain was inversely associated with falls (OR = 0.58, 95% CI: 0.35–0.97, *p* = 0.038). Table 5 summarizes the results for older adult pilgrims (aged ≥ 50 years).

## 4. Discussion

This study investigated the prevalence and predictors of falls among adult pilgrims during the Hajj using descriptive statistics, bivariate chi-square tests, and multivariable logistic regression analyses. The analyses were conducted for the overall sample and stratified by two physiologically distinct age groups: younger adults (aged ≤ 29 years) and older adults (aged ≥ 50 years). Bivariate analyses indicated that fall prevalence was significantly higher among females, older adults, and smokers. Clinical conditions such as hypertension and diabetes, along with physical exhaustion and overall musculoskeletal pain, were also associated with increased fall risk. In addition, several site-specific musculoskeletal pain regions, including the shoulder, upper arm, elbow, forearm, wrist/hand, head, cervical spine, thoracic spine, hip/pelvis, and knee, were significantly associated with falls. In the multivariable logistic regression models, obesity, hypertension, physical exhaustion, and musculoskeletal pain remained consistent predictors of falls. Age-stratified analyses further revealed that obesity, physical exhaustion, and upper arm pain were key predictors among younger adults, while obesity, hypertension, and hip/pelvis pain were more prominent among older adults. Notably, lumbar pain was inversely associated with falls in both the overall and older adult models, suggesting a potential protective or compensatory effect that warrants further investigation. These findings highlight the multifactorial nature of fall risk during Hajj, shaped by a complex interplay of demographic, behavioural/clinical, and musculoskeletal factors within an exceptionally demanding physical environment.

### 4.1. Fallers vs. Non-Fallers

The chi-square analysis revealed significant differences in demographic, clinical, and musculoskeletal variables between fallers and non-fallers. Fall prevalence was notably higher among older adults, which is consistent with established evidence linking age-related declines in muscle strength, sensory integration, and balance control to increased fall risk [14,16]. A higher rate of falls among females was also observed, aligning with previous studies that associate this pattern with lower muscle mass, hormonal influences on bone health, and a higher prevalence of musculoskeletal conditions that impair stability [17,18]. Smoking was more prevalent among fallers, supporting research that links tobacco use to vascular dysfunction and musculoskeletal degeneration, which may compromise mobility and postural stability [25]. Clinically, hypertension and diabetes were significantly associated with falls. These conditions are known to contribute to impaired proprioception, neuropathic symptoms, and fatigue, all of which increase susceptibility to instability and falls during physical exertion [18,28]. Physical exhaustion was reported more frequently by fallers, suggesting that fatigue reduces coordination, reaction time, and attentional capacity in physically demanding settings such as the Hajj [29,30]. Musculoskeletal pain, particularly in the hip/pelvis, knee, shoulder, upper arm, elbow, and spine, was also significantly associated with falls. These pain sites may alter gait patterns, disrupt load distribution, and impair postural control [24,35]. Although pain in the lower back and foot were not significantly associated with falls, observed trends may still have clinical implications and merit further investigation [13,26]. Taken together, these findings indicate that demographic factors, lifestyle behaviours, chronic conditions, fatigue, and localized musculoskeletal pain all contribute to fall risk, supporting the need for multivariable analysis to identify independent predictors.

### 4.2. Predictors of Falls: Overall Sample

Multivariable logistic regression identified several independent predictors of falls, underscoring the complex and multifactorial nature of fall risk among adult pilgrims. Older age was a significant predictor, corroborating existing literature on the association between aging, diminished neuromuscular function, and increased risk of imbalance and instability [14,16,17]. Obesity was also significantly linked to falls, likely due to its effects on gait mechanics, balance, and joint stress, particularly in weight-bearing regions such as the hips and knees [19,20,24,35]. Hypertension and diabetes were both significantly associated with increased fall risk. These chronic conditions can impair vascular integrity, proprioceptive feedback, and muscular endurance, thereby compromising postural control and increasing vulnerability to falls [18,28]. Notably, physical exhaustion emerged as the strongest overall predictor, indicating that fatigue-related impairments in motor coordination and attention substantially heighten fall risk during prolonged or intense physical activity [29,30]. Musculoskeletal pain in specific regions, including the upper arm, elbow, and hip/pelvis, was also independently associated with falls. Pain in these areas may disrupt normal arm swing, reduce trunk stability, or alter lower limb biomechanics, all of which affect balance during movement [24,27]. Interestingly, lumbar pain was inversely associated with falls, possibly reflecting behavioural adaptations such as reduced activity levels or cautious mobility in individuals with chronic lower back pain [26]. These findings support the development of integrated fall prevention strategies that address age-related decline, obesity, cardiometabolic health, fatigue, and region-specific musculoskeletal impairments in preparation for the physically demanding conditions of the Hajj.

### 4.3. Predictors of Falls in Younger Adults

Among younger adult pilgrims, fall risk was driven by different factors compared to the older group. Physical exhaustion was the most prominent predictor, highlighting the impact of sustained physical activity, heat exposure, and task-related strain during rituals. Fatigue is known to impair balance and reduce motor responsiveness, particularly in high-demand environments such as the Hajj [29,30]. Upper arm pain was also significantly associated with falls in this subgroup. This finding likely reflects localized overuse or musculoskeletal strain from repetitive upper limb activities such as lifting luggage or assisting others, which can impair protective reflexes and postural adjustments [24]. Although chronic conditions were not independently associated with falls in this age group, obesity remained a significant predictor. This reinforces previous findings that higher BMI negatively affects gait dynamics, contributes to early fatigue, and reduces postural stability in younger populations [20,22]. These observations emphasize the need for age-specific fall prevention measures for younger adults, focusing on physical preparedness, ergonomic awareness, and proactive fatigue management rather than clinical screening for chronic disease.

### 4.4. Predictors of Falls in Older Adults

In older adults, fall risk was influenced by a combination of metabolic, physical, and musculoskeletal factors. Obesity was independently associated with falls, supporting earlier findings that excessive body mass impairs gait stability and functional capacity [19,20,21]. Hypertension and diabetes were also significant predictors, consistent with evidence that links these comorbidities to impaired sensory feedback, muscular fatigue, and compromised postural control [18,28]. Physical exhaustion was a strong and consistent predictor of falls in this age group, further highlighting the role of fatigue in reducing neuromuscular coordination and increasing the likelihood of missteps or balance loss [29,30]. Hip and pelvic pain was also significantly associated with fall risk, likely due to its influence on gait mechanics and weight-bearing function [24]. Conversely, lumbar pain showed a negative association with falls. This may reflect self-regulating behaviours in individuals with chronic back pain, such as reduced ambulation or more cautious movement patterns, which could lower exposure to fall-inducing situations [26].

Prior literature has shown that individuals with chronic lumbar pain often adopt protective behavioural adaptations, such as decreased gait speed, limited bending, and avoidance of crowded environments [36,37]. These adaptations may unintentionally lower fall risk in mass gathering contexts. However, this was not a primary outcome of our analysis, and no sensitivity testing was conducted to evaluate the rigor of this finding. As such, the result should be interpreted cautiously and warrants further investigation in future studies.

Overall, the findings indicate that targeted fall prevention in older adults should include comprehensive management of obesity, hypertension, and diabetes, alongside interventions aimed at reducing fatigue and rehabilitating musculoskeletal impairments affecting the lower limbs and trunk.

### 4.5. Clinical Implications

The findings highlight the importance of individualized fall prevention strategies in clinical and public health settings, particularly for older adults with obesity, hypertension, or hip and pelvic pain. Health professionals should adopt comprehensive musculoskeletal assessments and implement evidence-based interventions such as physiotherapy, therapeutic exercise, and appropriate pain management to enhance mobility and reduce fall risk. In addition, routine screening for physical exhaustion during physically demanding activities like the Hajj pilgrimage may provide a timely method to identify individuals at increased risk of falling. Incorporating such assessments into pre-travel or on-site care can support early intervention and improve safety among vulnerable pilgrims.

These findings provide evidence for the development of targeted screening strategies before and during mass gatherings. Simple triage protocols may involve brief screening questions on physical exhaustion, upper limb or hip/pelvic pain, and chronic conditions such as hypertension or obesity. Pre-departure medical evaluations could also include fatigue-resistance or balance-related questions, footwear selection, and the safe use of umbrellas. Such triage systems would allow healthcare providers to efficiently allocate resources and facilitate support to at risk of falls. Contrary to clinical fall prevention, which may involve prolonged rehabilitation, mass-gathering strategies require rapid screening tools, environmental risk mitigation, and brief resource-efficient interventions delivered instantaneously within a highly dynamic and time-constrained setting. Our study supports this shift by identifying predictors that can be operationalized at scale using brief, non-invasive screening tools.

Although this study was conducted specifically during the Hajj, the physical and environmental stressors it addresses, such as prolonged walking, high crowd density, elevated temperatures, and musculoskeletal fatigue, are not exclusive to this religious pilgrimage. Similar risk profiles may be observed in other high-density travel settings, including major urban tourism destinations such as New York City, Rome, and Athens, as well as physically demanding pilgrimage routes like the Camino de Santiago in Spain. These shared characteristics suggest that the findings of this study may have broader external validity and could inform fall risk screening and prevention strategies in other global contexts where older adults and physically vulnerable individuals are exposed to prolonged walking and physical exertion under conditions of heat or crowding.

### 4.6. Recommendations

Future research should focus on designing and testing age-specific pre-Hajj screening and conditioning programs, particularly for older adults with multiple comorbidities and musculoskeletal pain. These programs should integrate cardiometabolic assessments, fatigue mitigation strategies, and targeted rehabilitation for high-risk joints such as the hip and knee. For younger adults, future studies should investigate interventions that address physical preparedness, upper limb strain, and fatigue management. The potential utility of wearable activity monitors and standardized functional tests, such as gait and balance assessments, also warrants exploration to support real-time fall risk monitoring in high-exertion environments. Additionally, the inverse association observed between low back pain and falls requires deeper investigation. This unexpected relationship may reflect protective behaviours or compensatory movement strategies. Longitudinal and mixed-methods research is needed to examine these hypotheses and to evaluate the long-term effectiveness of multifactorial interventions across different risk profiles.

### 4.7. Limitations

There are some limitations to consider in this study. First, the study employed a convenience sampling approach, which may introduce selection bias and limit the representativeness of the findings. Although random or probabilistic sampling was not feasible in the Hajj environment due to safety, crowd-control, and logistical constraints, several measures were undertaken to reduce potential bias, including recruitment across multiple sites, time windows, and demographic strata using standardized scripts. Accordingly, the findings should be interpreted as event-based inferences within the Hajj context rather than as population-level estimates. In addition, participants’ country and region of residence were not included in the analyses, which may limit interpretation of cultural or contextual influences. Future investigations should consider employing probability-based or stratified sampling and incorporating such demographic variables into analytical models to enhance external validity and generalizability of results. Second, the exclusion of the 30–49-year age group and the absence of data on fall frequency or severity may limit the interpretation of fall risk patterns across the aging spectrum. Accordingly, comparisons between fallers and non-fallers should be interpreted with caution, as observed differences may partly reflect the analytical focus on the two extreme age groups. Nevertheless, the inclusion of younger (≤29 years) and older (≥50 years) pilgrims was intended to emphasize physiologically distinct risk profiles and maintain analytical clarity within an event-based design. Future investigations should consider incorporating intermediate age groups and collecting detailed information on fall frequency and severity to enable a more comprehensive understanding of how fall risk evolves across age groups. Third, calculating a precise response rate was not feasible due to the dynamic flow of pilgrims, safety protocols, and limited site access during the Hajj mass gathering. This constraint may have introduced nonresponse bias and should be acknowledged when interpreting the findings. Nonetheless, field teams documented eligible and consented participants, ineligible cases, and incomplete surveys where feasible, and these counts are summarized in the participant flow chart. Despite this operational limitation, the large and diverse sample obtained across multiple sites supports the robustness and practical value of the study’s findings. Fourth, all collected data were self-reported by participants, which may introduce recall or reporting bias and variability in how terms such as ‘pain,’ ‘exhaustion,’ or ‘falls’ were interpreted. Although self-reporting represents a practical and widely used approach in large-scale, real-world field studies such as Hajj, the absence of objective verification may affect the accuracy of some variables. The operational definition of falls used in the questionnaire was intentionally broad to capture a wide range of relevant events during pilgrimage; however, this may have included minor, non-physiological incidents and should be considered when interpreting results. Future studies should incorporate validated instruments, standardized definitions, and objective or externally verified measures where feasible to improve the reliability and validity of findings. Fifth, given the number of predictors examined in both chi-square and regression analyses, there is an increased risk of Type I error. Although no formal correction for multiple comparisons was applied, the study is exploratory in nature, and multivariable models were used to account for confounding. Although a large number of bivariate comparisons were conducted, increasing the likelihood of Type I error, we did not apply a Bonferroni correction due to the exploratory nature of the analysis. This decision was partially mitigated by follow-up multivariable regression, which provided adjusted estimates of association. Sixth, the cross-sectional design limits the ability to determine cause-and-effect relationships between the identified factors and fall outcomes. Future longitudinal or interventional studies are needed to establish temporal and causal associations. Finally, the data were collected during the 2018 Hajj season, which may not fully capture recent contextual or environmental changes. Nevertheless, as the structure and physical demands of Hajj rituals have remained largely consistent, and no subsequent studies have investigated similar research questions in this context, these findings continue to offer relevant insights into musculoskeletal and fall-related risks in this unique mass-gathering setting.

## 5. Conclusions

This study provides novel age-stratified insights into fall risk among adult pilgrims during the Hajj mass gathering, highlighting distinct predictors such as hypertension and hip pain among older adults and upper arm pain among younger adults. Physical exhaustion emerged as the most consistent and potentially modifiable predictor across all groups, underscoring the importance of fatigue screening and mitigation during physically demanding mass gatherings. These findings contribute to the growing literature on mass-gathering health and support the development of scalable screening protocols for identifying at-risk individuals in real time. However, the interpretation of associations must be made with caution. The reliance on self-reported data may introduce recall or reporting bias, particularly regarding comorbidities and fall events. Additionally, the use of convenience sampling limits the representativeness of the findings. Despite these limitations, the study offers a foundation for future longitudinal research and public health interventions targeting fall prevention in high-risk, high-mobility environments such as Hajj.

## Figures and Tables

**Table 1:** Characteristics and health-related variables of younger and older adult pilgrims.

Variable	Age ≤ 29 Years		Age ≥ 50 Years		*p* Value
	Count	Prev.% (95% CI)	Count	Prev.% (95% CI)	
Male	557	71.6% (68.0–74.9)	221	28.4% (25.1–32.0)	0.894
Female	464	71.3% (67.5–74.8)	187	28.7% (25.2–32.5)	
BMI < 25 kg/m^2^	608	59.5% (56.5–62.5)	122	30% (25.7–34.5)	**˂0.001**
BMI ≥ 30 kg/m^2^	215	21.1% (18.7–23.7)	175	43% (38.2–47.7)	
Smoker	224	22% (19.5–24.6)	92	22.5% (18.8–26.8)	0.802
Non-smoker	797	78% (75.4–80.5)	316	77.5% (73.2–81.2)	
Participants with (yes):					
Hypertension	66	6.5% (5.1–8.1)	175	43% (38.2–47.7)	**˂0.001**
Diabetes	64	6% (4.9–7.9)	136	33% (28.9–38.0)	**˂0.001**
Physical exhaustion	410	40% (37.2–43.2)	230	56.4% (51.5–61.1)	**˂0.001**
Overall musculoskeletal pain	810	79% (76.7–81.7)	358	88% (84.2–90.6)	**˂0.001**
Shoulder pain	162	16% (13.8–18.2)	70	17% (13.8–21.1)	0.578
Upper arm pain	32	3% (2.2–4.4)	14	3.5% (2.1–5.7)	0.774
Elbow pain	22	2% (1.4–3.2)	19	5% (3.0–7.2)	**0.010**
Forearm pain	24	2.5% (1.6–3.5)	21	5% (3.4–7.7)	**0.006**
Wrist/Hand pain	36	3.5% (2.6–4.8)	32	8% (5.6–10.9)	**˂0.001**
Head pain	153	15% (12.9–17.30)	67	16.5% (13.1–20.3)	0.497
Cervical pain	127	12.5% (10.6–14.6)	63	15.5% (12.3–19.3)	0.131
Thoracic pain	136	13% (11.4–15.5)	64	16% (12.5–19.5)	0.272
Lumbar pain	274	27% (24.2–29.6)	408	31.5% (27.1–36.0)	0.085
Hip/Pelvis pain	41	4% (3.0–5.4)	45	11% (8.3–14.4)	**˂0.001**
Thigh pain	152	15% (12.8–17.2)	49	12% (9.2–15.5)	0.158
Knee pain	158	15.5% (13.4–17.8)	165	40.5% (35.8–45.3)	**˂0.001**
Leg pain	291	28.5% (25.8–31.3)	145	35.5% (31.0–40.3)	**0.009**
Ankle/Foot pain	423	41.5% (38.4–44.5)	147	36% (31.5–40.8)	0.060

BMI: body mass index; Prev.: prevalence percent; CI: confidence interval. Frequencies (counts) and prevalence estimates (percentages with 95% confidence intervals) are reported. *p*-values were derived from chi-square tests, and statistically significant differences between age groups (*p* < 0.05) are presented in bold.

**Table 2:** Bivariate chi-square analysis of fall prevalence among all adult pilgrims.

Variable	Fallers		Non-Fallers		*p* Value
	Count	Prev.% (95% CI)	Count	Prev.% (95% CI)	
Male	90	11.6% (9.3–13.8)	688	88.4% (86.2–90.7)	**0.015**
Female	105	16.1% (13.3–19.0)	546	83.9% (81.0–86.7)	
Age ≤ 29 years	108	10.5% (8.9–12.7)	913	89% (87.4–91.2)	**<0.001**
Age ≥ 50 years	87	21% (17.6–25.5)	321	79% (74.4–82.4)	
BMI < 25 kg/m^2^	109	14.9% (12.3–17.5)	621	85.1% (82.5–87.7)	0.217
BMI ≥ 30 kg/m^2^	47	12.1% (8.8–15.3)	343	87.9% (84.7–90.2)	
Smoker	49	15.5% (11.5–19.5)	267	84.5% (80.5–88.5)	0.318
Non-smoker	146	13.1% (11.1–15.1)	967	86.9% (84.9–88.9)	
Participants with (yes):					
Hypertension	84	27% (22.4–32.2)	152	11% (9.3–12.6)	**<0.001**
Diabetes	68	28% (22.4–33.5)	178	72% (66.5–77.6)	**<0.001**
Physical exhaustion	159	21% (18.2–24.0)	603	79% (76.0–81.8)	**<0.001**
Overall musculoskeletal pain	221	16% (13.9–17.7)	1190	84% (82.3–86.1)	**<0.001**
Shoulder pain	58	21% (16.6–26.2)	218	79% (73.8–83.4)	**<0.001**
Upper arm pain	20	35% (24.0–48.1)	37	65% (51.9–76.0)	**<0.001**
Elbow pain	18	38% (26.1–52.1)	29	62% (47.9–73.9)	**<0.001**
Forearm pain	13	26% (15.8–39.5)	37	74% (60.5–84.2)	**0.011**
Wrist/Hand pain	23	29% (20.3–39.7)	56	71% (60.3–79.7)	**<0.001**
Head pain	50	19% (14.6–23.8)	216	81% (76.2–85.4)	**0.009**
Cervical pain	47	20.5% (15.8–26.2)	182	79.5% (73.8–84.2)	**0.001**
Thoracic pain	46	20% (15.7–26.0)	179	80% (74.0–84.3)	**0.002**
Lumbar pain	66	14% (10.8–16.9)	421	86% (83.1–89.2)	0.875
Hip/Pelvis pain	29	30% (21.5–39.3)	69	71% (60.7–78.5)	**<0.001**
Thigh pain	38	16.5% (12.2–21.7)	194	83.5% (78.3–87.8)	0.213
Knee pain	72	18% (14.7–22.3)	323	82% (77.7–85.3)	**0.003**
Leg pain	79	15% (12.3–18.5)	442	85% (81.5–87.7)	0.265
Ankle/Foot pain	103	15.5% (12.8–18.3)	567	84.5% (81.7–87.2)	0.121

BMI: body mass index; Prev.: prevalence percent; CI: confidence interval. Frequencies (counts) and prevalence estimates (percentages with 95% confidence intervals) are reported. *p*-values were derived from chi-square tests, and statistically significant differences between fallers and non-fallers (*p* < 0.05) are presented in bold.

**Table 3:** Multivariable logistic regression analysis predicting fall prevalence among all adult pilgrims.

Predictor	B (SE)	OR (95% CI)	*p* Value
Sex (female vs. male)	−0.37 (0.19)	0.69 (0.48–1.00)	0.051
Age group (old vs. young)	0.75 (0.15)	2.11 (1.57–2.82)	**<0.001**
BMI (≥30 vs. <25 kg/m^2^)	0.61 (0.19)	1.84 (1.26–2.69)	**0.002**
Smoking (smoker vs. non-smoker)	0.29 (0.22)	1.33 (0.87–2.03)	0.185
Participants with (yes vs. no):			
Hypertension	0.84 (0.21)	2.31 (1.54–3.45)	**<0.001**
Diabetes	0.64 (0.22)	1.90 (1.24–2.91)	**0.003**
Physical exhaustion	1.04 (0.19)	2.83 (1.96–4.07)	**<0.001**
Overall musculoskeletal pain	0.45 (0.34)	1.57 (0.81–3.04)	0.178
Shoulder pain	−0.15 (0.23)	1.16 (0.75–1.81)	0.506
Upper arm pain	0.77 (0.38)	2.16 (1.03–4.56)	**0.042**
Elbow pain	1.42 (0.31)	4.13 (2.25–7.57)	**<0.001**
Forearm pain	0.08 (0.45)	1.08 (0.45–2.58)	0.863
Wrist/Hand pain	0.37 (0.35)	1.45 (0.73–2.87)	0.289
Head pain	0.12 (0.22)	1.13 (0.73–1.74)	0.591
Cervical pain	−0.15 (0.25)	0.86 (0.53–1.40)	0.542
Thoracic pain	−0.12 (0.24)	0.89 (0.56–1.40)	0.623
Lumbar pain	−0.46 (0.20)	0.63 (0.42–0.94)	**0.023**
Hip/Pelvis pain	0.74 (0.30)	2.10 (1.17–3.79)	**0.013**
Thigh pain	−0.12 (0.25)	0.88 (0.55–1.43)	0.614
Knee pain	0.15 (0.20)	1.16 (0.79–1.71)	0.453
Leg pain	−0.08 (0.19)	0.93 (0.64–1.35)	0.685
Ankle/Foot pain	0.03 (0.18)	1.03 (0.72–1.47)	0.880

B: regression coefficient; SE: standard error; OR: odds ratio; CI: confidence interval. Regression coefficients with standard errors, odds ratios, and corresponding 95% confidence intervals are reported. *p*-values were derived from multivariable binary logistic regression analysis, and statistically significant predictors of falls among all adult pilgrims (*p* < 0.05) are presented in bold.

**Table 4:** Multivariable logistic regression analysis predicting fall prevalence among younger adult pilgrims.

Predictor	B (SE)	OR (95% CI)	*p* Value
Sex (female vs. male)	−0.12 (0.35)	0.89 (0.46–1.73)	0.734
BMI (≥30 vs. <25 kg/m^2^)	0.72 (0.34)	2.05 (1.06–3.95)	**0.033**
Smoking (smoker vs. non-smoker)	−0.32 (0.40)	0.73 (0.33–1.59)	0.427
Participants with (yes vs. no):			
Hypertension	0.55 (0.34)	1.73 (0.89–3.36)	0.106
Diabetes	0.29 (0.34)	1.33 (0.69–2.59)	0.396
Physical exhaustion	1.54 (0.41)	4.66 (2.10–10.36)	**<0.001**
Overall musculoskeletal pain	18.88 (6444.78)	158,147,952.9 (≈0–∞)	0.998
Shoulder pain	0.21 (0.43)	1.24 (0.54–2.86)	0.616
Upper arm pain	2.51 (0.92)	12.26 (2.06–72.90)	**0.007**
Elbow pain	0.48 (0.74)	1.62 (0.39–6.83)	0.514
Forearm pain	−0.21 (0.74)	0.81 (0.19–3.47)	0.780
Wrist/Hand pain	−0.21 (0.58)	0.81 (0.26–2.49)	0.710
Head pain	0.57 (0.43)	1.76 (0.76–4.07)	0.186
Cervical pain	−0.25 (0.44)	0.78 (0.33–1.85)	0.576
Thoracic pain	0.06 (0.41)	1.06 (0.47–2.39)	0.887
Lumbar pain	−0.36 (0.36)	0.70 (0.35–1.40)	0.313
Hip/Pelvis pain	0.31 (0.49)	1.37 (0.53–3.56)	0.519
Thigh pain	−0.22 (0.47)	0.81 (0.32–2.03)	0.644
Knee pain	0.59 (0.34)	1.81 (0.94–3.47)	0.078
Leg pain	−0.46 (0.34)	0.63 (0.32–1.23)	0.168
Ankle/Foot pain	0.51 (0.33)	1.67 (0.87–3.19)	0.139

B: regression coefficient; SE: standard error; OR: odds ratio; CI: confidence interval. Regression coefficients with standard errors, odds ratios, and corresponding 95% confidence intervals are reported. *p*-values were derived from multivariable binary logistic regression analysis, and statistically significant predictors of falls among younger adult pilgrims (*p* < 0.05) are presented in bold.

**Table 5:** Multivariable logistic regression analysis predicting fall prevalence among older adult pilgrims.

Predictor	B (SE)	OR (95% CI)	*p* Value
Sex (female vs. male)	−0.45 (0.24)	0.64 (0.40–1.00)	0.053
BMI (≥30 vs. <25 kg/m^2^)	0.81 (0.27)	2.24 (1.31–3.85)	**0.003**
Smoking (smoker vs. non-smoker)	0.53 (0.27)	1.69 (1.00–2.86)	0.052
Participants with (yes vs. no):			
Hypertension	0.83 (0.29)	2.28 (1.26–4.13)	**0.010**
Diabetes	0.57 (0.33)	1.77 (0.93–3.34)	0.081
Physical exhaustion	0.88 (0.22)	2.40 (1.56–3.70)	**<0.001**
Overall musculoskeletal pain	0.27 (0.36)	1.31 (0.65–2.65)	0.452
Shoulder pain	0.24 (0.28)	1.27 (0.73–2.22)	0.394
Upper arm pain	0.39 (0.47)	1.48 (0.59–3.73)	0.499
Elbow pain	0.72 (0.55)	2.04 (0.70–5.95)	0.191
Forearm pain	0.22 (0.62)	1.25 (0.37–4.17)	0.720
Wrist/Hand pain	0.51 (0.45)	1.66 (0.68–4.04)	0.221
Head pain	0.04 (0.28)	1.04 (0.61–1.79)	0.881
Cervical pain	−0.16 (0.32)	0.85 (0.46–1.60)	0.624
Thoracic pain	−0.19 (0.33)	0.83 (0.43–1.59)	0.581
Lumbar pain	−0.54 (0.26)	0.58 (0.35–0.97)	**0.038**
Hip/Pelvis pain	0.98 (0.39)	2.66 (1.23–5.74)	**0.013**
Thigh pain	0.02 (0.30)	1.02 (0.57–1.83)	0.948
Knee pain	−0.27 (0.28)	0.76 (0.44–1.33)	0.301
Leg pain	0.05 (0.25)	1.05 (0.65–1.70)	0.842
Ankle/Foot pain	−0.18 (0.23)	0.84 (0.53–1.32)	0.839

B: regression coefficient; SE: standard error; OR: odds ratio; CI: confidence interval. Regression coefficients with standard errors, odds ratios, and corresponding 95% confidence intervals are reported. *p*-values were derived from multivariable binary logistic regression analysis, and statistically significant predictors of falls among older adult pilgrims (*p* < 0.05) are presented in bold.

## Data Availability

The original contributions presented in the study are included in the article; further inquiries can be directed at the corresponding author.

## Abbreviations

The following abbreviations are used in this manuscript:

WHO	World Health Organization
BMI	Body mass index
STROBE	Strengthening the Reporting of Observational Studies in Epidemiology
95% CI	95% confidence interval
B	Regression coefficient
SE	Standard error
OR	Odds ratio
Prev.	Prevalence percent

## References

[1] Memish Z.A., Zumla A., Alhakeem R.F., Assiri A., Turkestani A., Al Harby K.D., Alyemni M., Dhafar K., Gautret P., Barbeschi M. (2014). Hajj: Infectious disease surveillance and control. Lancet.

[2] Shafi S.S.S., Dar O.D., Khan M.K.M., Azhar E.I., McCloskey B., Zumla A., Petersen E. (2016). The annual Hajj pilgrimage—Minimizing the risk of ill health in pilgrims from Europe. Euro Surveill..

[3] Madani T.A., Ghabrah T.M., Al-Hedaithy M.A., Alhazmi M.A., Alazraqi T.A., Albarrak A.M., Ishaq A.H. (2006). Causes of hospitalization of pilgrims during the Hajj period of the Islamic year 1423 (2003). Ann. Saudi Med..

[4] Samarkandi O., Alamri F., Alsaleh G., Al Abdullatif L., Alhazmi J., Basnawi M., Alazmy W., Khan A. (2025). Exploring the prevalence of chronic diseases and health status among international Hajj pilgrims. PLoS ONE.

[5] Sridhar S., Benkouiten S., Belhouchat K., Drali T., Memish Z.A., Parola P., Brouqui P., Gautret P. (2015). Foot ailments during Hajj: A short report. J. Epidemiol. Glob. Health.

[6] Alshehri M.A., Alzaidi J., Alasmari S., Alfaqeh A., Arif M., Alotaiby S.F., Alzahrani H. (2021). The prevalence and factors associated with musculoskeletal pain among pilgrims during the Hajj. J. Pain Res..

[7] Koubâa A., Ammar A., Benjdira B., Al-Hadid A., Kawaf B., Al-Yahri S.A., Babiker A., Assaf K., Ras M.B. Activity monitoring of Islamic prayer (salat) postures using deep learning. Proceedings of the 2020 6th International Conference on Control, Decision and Information Technologies (CoDIT).

[8] Alqahtani A.S., Tashani M., Heywood A.E., Almohammed A.B.S., Booy R., Wiley K.E., Rashid H. (2020). Tracking Australian Hajj pilgrims’ health behavior using a mobile app. Pharmacy.

[9] Kolivand P., Saberian P., Saffari H., Doroudi T., Marashi A., Behzadifar M., Karimi F., Rajaei S., Raei B., Ehsanzadeh S.J. (2024). Patterns of diabetes mellitus among Iranian Hajj pilgrims: A nationwide cross-sectional study. PLoS ONE.

[10] Aldossari M., Aljoudi A., Celentano D. (2019). Health issues in the Hajj pilgrimage: A literature review. East. Mediterr. Health J..

[11] Kanungo S., Bhattacharjee U., Prabhakaran O.A., Kumar R., Rajkumar P., Bhardwaj S.D., Chakrabarti A.K., Kumar CP G., Potdar V., Manna B. (2024). Adverse outcomes in patients hospitalized with pneumonia during the Hajj. PLoS ONE.

[12] Alamri F.A., Amer S.A., Alhraiwil N.J. (2018). Knowledge and practice after health education program among Hajj pilgrims. Saudi Arabia J. Epidemiol. Health Care.

[13] Alghamdi G.A., Alghamdi F.A., Almatrafi R.M., Sadis A.Y., Shabkuny R.A., Alzahrani S.A., Alessa M.Q., Hafiz W.A., Shabkuny R., Alessa M.Q. (2024). The prevalence of musculoskeletal injuries among pilgrims during the 2023 Hajj season: A cross-sectional study. Cureus.

[14] Salari N., Darvishi N., Ahmadipanah M., Shohaimi S., Mohammadi M. (2022). Global prevalence of falls in the older adults: A systematic review and meta-analysis. J. Orthop. Surg. Res..

[15] World Health Organization (2024). World Health Statistics 2024: Monitoring Health for the SDGs.

[16] Adam C.E., Fitzpatrick A.L., Leary C.S., Ilango S.D., Phelan E.A., Semmens E.O. (2024). The impact of falls on activities of daily living in older adults: Longitudinal evidence from a population-based study. PLoS ONE.

[17] Giovannini S., Brau F., Galluzzo V., Santagada D.A., Loreti C., Biscotti L., Laudisio A., Zuccala G., Bernabei R. (2022). Falls among older adults: Screening, identification, rehabilitation, and management. Appl. Sci..

[18] Freire L.B., Brasil-Neto J.P., da Silva M.L., Miranda M.G.C., de Mattos Cruz L., Martins W.R., da Silva Paz L.P. (2024). Risk factors for falls in older adults with diabetes mellitus: A population-based study. BMC Geriatr..

[19] Villareal D.T., Apovian C.M., Kushner R.F., Klein S. (2005). Obesity in older adults: Technical review and position statement of the American Society for Nutrition. Am. J. Clin. Nutr..

[20] Sheehan K.J., O’Connell M.D.L., Cunningham C., Crosby L., Kenny R.A. (2013). The relationship between increased body mass index and frailty on falls in community dwelling older adults. BMC Geriatr..

[21] Stenholm S., Strandberg T.E., Pitkälä K., Sainio P., Heliövaara M., Koskinen S. (2014). Midlife obesity and risk of frailty and disability in later life: A 22-year follow-up study. J. Gerontol. A Biol. Sci. Med. Sci..

[22] Watanabe D., Yoshida T., Watanabe Y., Yamada Y., Kimura M. (2025). Body mass index and survival with disability among community-dwelling older adults. Int. J. Obes..

[23] Sun Q., Xia X., He F. (2024). Body mass index and frailty risk: A systematic review and meta-analysis. Arch. Gerontol. Geriatr..

[24] Tiwari J., Halder P., Sharma D., Saini U.C., Rajagopal V., Kiran T. (2024). Prevalence and risk factors for musculoskeletal disorders among adults: A cross-sectional study. PLoS ONE.

[25] Jeong J., Yoshimoto T., Suganuma A., Nakata A. (2023). Tobacco and alcohol consumption and the risk of frailty and falling: A prospective study of middle-aged and older adults in Japan. J. Epidemiol. Community Health.

[26] Jawad N.A.M., Alosami M.H., Mahdi Z.F. (2024). Chronic low back pain and quality of life in Iraq. Med. J. Babylon.

[27] Welsh V.K., Clarson L.E., Mallen C.D., McBeth J. (2019). Multisite pain and falls in older people: A systematic review. Arthritis Res. Ther..

[28] Ma L., Zhang L., Sun F., Li Y., Tang Z. (2018). Frailty in Chinese older adults with hypertension and its association with falls. J. Clin. Hypertens..

[29] Zhang Q., Zhao X., Liu H., Ding H. (2020). Frailty as a predictor of future falls and disability in older adults. BMC Geriatr..

[30] Abou L., Fritz N.E., Kratz A.L. (2023). Fatigue and injurious falls in patients with multiple sclerosis. Mult. Scler. Relat. Disord..

[31] Lee Y.C., Chang S.F., Kao C.Y., Tsai H.C. (2022). Balance and walking ability at fall risk in prefrail older people. Biomed. Res. Int..

[32] Erlandson K.M., Allshouse A.A., Jankowski C.M., Duong S., MaWhinney S., Kohrt W.M., Campbell T.B. (2012). Risk factors for falls in HIV-infected persons. J. Acquir. Immune Defic. Syndr..

[33] Al-Hayani M.M., Kamel S., Al-Hayani A.M., Al-Hazmi E.A., Al-Shanbari M.S., Al-Otaibi N.S., Almeshal A.S., Assiri A.M., Kamel S., Al-Hayani A. (2023). Trauma and injuries pattern during Hajj, 1443 (2022): A cross-sectional study. Cureus.

[34] Kolivand P., Saberian P., Arabloo J., Namdar P., Doroudi T., Marashi A., Behzadifar M., Karimi F., Rajaei S., Raei B. (2025). Hospitalization, mortality, and health service delivery pattern among Iranian Hajj pilgrims by age, sex, and province in 2013–22. Front. Public Health.

[35] Peplow P.V. (2024). Reprogramming T cells as an emerging treatment to slow human age-related decline in health. Front. Med. Technol..

[36] Bordeleau M., Vincenot M., Lefevre S., Duport A., Seggio L., Breton T., Lelard T., Serra E., Roussel N., Neves J.F.D. (2022). Treatments for kinesiophobia in people with chronic pain: A scoping review. Front. Behav. Neurosci..

[37] Ogawa E.F., Shi L., Bean J.F., Hausdorff J.M., Dong Z., Manor B., McLean R.R., Leveille S.G. (2020). Chronic pain characteristics and gait in older adults: The MOBILIZE Boston Study II. Arch. Phys. Med. Rehabil..

